# Exposure of Rats to Environmental Tobacco Smoke during Cerebellar Development Alters Behavior and Perturbs Mitochondrial Energetics

**DOI:** 10.1289/ehp.1104857

**Published:** 2012-09-26

**Authors:** Brian F. Fuller, Diego F. Cortes, Miranda K. Landis, Hiyab Yohannes, Hailey E. Griffin, Jillian E. Stafflinger, M. Scott Bowers, Mark H. Lewis, Michael A. Fox, Andrew K. Ottens

**Affiliations:** 1 Department of Anatomy and Neurobiology, and; 2 Department of Biochemistry and Molecular Biology, Virginia Commonwealth University, Richmond, Virginia, USA; 3 Department of Psychiatry, Virginia Institute of Psychiatric and Behavioral Genetics, Virginia Commonwealth University, Richmond, Virginia, USA; 4 Department of Psychiatry, McKnight Brain Institute of the University of Florida, Gainesville, Florida, USA

**Keywords:** attention deficit/hyperactivity disorder, carbohydrate metabolism, cerebellum, environmental tobacco smoke, mitochondrial biogenesis, mitochondrial energetics, neurodevelopment, proteomics, secondhand smoke, systems biology

## Abstract

Background: Environmental tobacco smoke (ETS) exposure is linked to developmental deficits and disorders with known cerebellar involvement. However, direct biological effects and underlying neurochemical mechanisms remain unclear.

Objectives: We sought to identify and evaluate underlying neurochemical change in the rat cerebellum with ETS exposure during critical period development.

Methods: We exposed rats to daily ETS (300, 100, and 0 µg/m^3^ total suspended particulate) from postnatal day 8 (PD8) to PD23 and then assayed the response at the behavioral, neuroproteomic, and cellular levels.

Results: Postnatal ETS exposure induced heightened locomotor response in a novel environment on par initially with amphetamine stimulation. The cerebellar mitochondrial subproteome was significantly perturbed in the ETS-exposed rats. Findings revealed a dose-dependent up-regulation of aerobic processes through the modification and increased translocation of Hk1 to the mitochondrion with corresponding heightened ATP synthase expression. ETS exposure also induced a dose-dependent increase in total Dnm1l mitochondrial fission factor; although more active membrane-bound Dnm1l was found at the lower dose. Dnm1l activation was associated with greater mitochondrial staining, particularly in the molecular layer, which was independent of stress-induced Bcl-2 family dynamics. Further, electron microscopy associated Dnm1l-mediated mitochondrial fission with increased biogenesis, rather than fragmentation.

Conclusions: The critical postnatal period of cerebellar development is vulnerable to the effects of ETS exposure, resulting in altered behavior. The biological effect of ETS is underlain in part by a Dnm1l-mediated mitochondrial energetic response at a time of normally tight control. These findings represent a novel mechanism by which environmental exposure can impact neurodevelopment and function.

Recent epidemiological studies find a dose-dependent increased risk for behavioral and cognitive problems and a greater incidence of mental disorders in children exposed to environmental tobacco smoke (ETS) (Anderko et al. 2010; Bandiera et al. 2011; Kabir et al. 2011). Confirming earlier findings, these studies addressed two major concerns highlighted in the U.S. Surgeon General report on ETS health consequences by employing objective biomarker measurements and determining that effects are independent of maternal smoking (U.S. Department of Health and Human Services 2006). Thus, nearly one in five U.S. children are at greater risk for mental health problems because of postnatal ETS exposure, a prevalence that has remained unchanged for over a decade (Centers for Disease Control and Prevention 2010). ETS exposure is more pronounced in the young because of their higher respiration rates and remains prevalent because 50% of mothers who cease smoking during pregnancy resume < 6 months after delivery (Colman and Joyce 2003; Marano et al. 2009). The issue is of even greater concern worldwide, with over 50% of children regularly exposed to ETS across large parts of Europe and Asia (Oberg et al. 2011). Yet, it remains undetermined whether early ETS exposure directly affects neurodevelopment to induce behavioral change and what biological mechanisms might underlie its effects.

Activity, attention, impulsivity, and language deficits, which have been reported with greater incidence in ETS-exposed children, all involve cerebellar regulation through feedback loops to the neocortex (Bledsoe et al. 2011; O’Halloran et al. 2012; Richter et al. 2005). Reduced cerebellar size and function have also been associated with attention deficit hyperactivity disorder (ADHD) and conduct disorders (Aguiar et al. 2010; Dalwani et al. 2011; O’Halloran et al. 2012; Richter et al. 2005). A meta-analysis of ADHD structural imaging studies found reproducible cerebellar abnormalities in areas of the posterior cerebellum, such as in lobule VIII (Valera et al. 2007). These results suggest cerebellar vulnerability that may be linked with its late development in mammals, including humans. Research by Dobbing and colleagues established the precept of a vulnerable period for neurodevelopment as reviewed elsewhere (Dobbing 1982), which in the human cerebellar cortex extends > 1 year after birth, rendering it susceptible to the effects of postnatal ETS (Dobbing 1982; Friede 1973; Koop et al. 1986). Corresponding rat cerebellar cortex development extends approximately between postnatal day (PD) 8 and PD24 (Altman and Bayer 1997; Gramsbergen 1993) and has been shown to be vulnerable to various insults, with lasting morphological and functional deficits (Altman and Bayer 1997; Bedi et al. 1980; Dobbing 1982). In the present study, we exposed rat pups to daily ETS [300, 100, and 0 µg/m^3^ total suspended particulate (TSP)] during the cerebellar vulnerable period, a rational initial point of investigation given its postnatal vulnerability and functional relevance to reported deficits and disorders. More broadly, this study *a*) addresses a lack of knowledge of the neurobiological effects of ETS during development, and *b*) studies potential mechanistic underpinnings.

## Materials and Methods

*Animal procedures and tissue collection*. Animals were treated humanely and with regard for alleviation of suffering. All procedures conformed to the U.S. Public Health Service policy with local institutional animal care and use committee approval. Pregnant Sprague-Dawley rats were purchased from Harlan Laboratories (Indianapolis, IN) and housed in a facility approved by the Association for Assessment and Accreditation of Laboratory Animal Care on a 12-hr light cycle with *ad libitum* access to food and water. We treated male rat pups daily from age PD8 to PD23 in a Teague TE-10 smoking system (Teague Enterprises, Woodland, CA) operated as described previously (Fuller et al. 2010; Gospe et al. 1996; Slotkin et al. 2001), with TSP levels confirmed daily. The first of two exposure groups received amplified ETS at a mean daily level of 300 µg/m^3^ TSP (ETS_300_), with peak concentrations of 2 mg/m^3^ during active smoking. The extreme concentration modeled here, which is realistic to ETS in cars (Ott et al. 2008), was used to more readily detect a biochemical response in our initial mechanistic studies. Exaggerated chronic exposure may also be considered relevant for ETS exposure in combination with urban pollution, where mean daily TSP levels can measure in large metropolitan areas in the hundreds of micrograms per cubic meter, principally from other combustion sources (Calderón-Garcidueñas et al. 2008). In a second exposure, we modeled upper quartile ETS levels found in homes with smokers, with a mean daily level of 100 µg/m^3^ TSP (ETS_100_) peaking at 0.5 mg/m^3^ during active smoking (U.S. Environmental Protection Agency 1992). This exposure approximated ETS levels recorded in bedrooms of preschool children in homes with a pack-a-day smoker (McCormack et al. 2008). Litters cumulatively received 3 hr/day of ETS exposure, apart from dams, with feedings in between. Control animals were handled identically except for not receiving ETS exposure. Mean daily carbon monoxide levels remained < 5 ppm. Animal weight was monitored daily and no difference was found between groups. Brains were collected after the close of the initial cerebellar vulnerability period at PD25.

*Locomotor activity.* Using previously described procedures (Stohr et al. 1998) with the following modifications, we measured spontaneous locomotor activity in an unfamiliar environment at PD25 during the light cycle (1100–1400 hours). Animals were placed in a 42 × 42 × 30 cm^3^ open field arena under red light illumination (1 lux at height of animal). Chambers were located within a sound-isolated room. ETS_100_-exposed animals and one control (Cnt) group received acute saline [Sal; 1 mL/kg, intraperitoneally (ip)] while a second, hyperlocomotor-positive control group received acute amphetamine (Amp; 1 mg/kg, ip): ETS/Sal, Cnt/Sal, Cnt/Amp. We placed animals in the center of the arena 15 min after injection and recorded activity (total distance traveled, maximum velocity, and entries into a 14 × 14 cm^2^ central zone) assessed in 1-min intervals using ANY-maze tracking software (version 4.84; Stoelting, Wood Dale, IL). We cleaned the arena with 90% ethanol with the odor blown off before subsequent testing. The 1-mg/kg amphetamine dose was based on our pilot data indicating the dose would increase locomotor activity without stereotypic behavior. In contrast, both increased locomotion and stereotypic behavior were observed at a 3-mg/kg dose level (data not shown).

*Two-dimensional chromatography-tandem mass spectrometry*. Tissue from the cerebellar hemisphere was processed through a multistep protein extraction procedure described previously (Cortes et al. 2012). Briefly, we sequentially homogenized tissue in aqueous and membrane dissociation buffers (matrix and membrane extracts, respectively) to resolve the neuroproteome into matrix-associated and membrane-associated compartments (Cortes et al. 2012). We assayed protein concentration with a Pierce 660 kit (Thermo Scientific, Rockford, IL). Protein samples (50 μg) were reduced, alkylated, trypsin-digested, and concentrated into 20 µL of 100-mM ammonium formate (pH 10). We injected protein digests (4 µL each) in a treatment-interspersed order onto a two-dimensional nanoACQUITY ultra performance liquid chromatography system using an On-Line RP/RP 2D Separations kit ahead of a Synapt HDMS mass spectrometer operated in a data-independent acquisition mode (all from Waters, Milford, MA). We used Waters PLGS software (version 2.4) to process and annotate mass spectral data [using the Uniprot KB Rattus database (http://www.uniprot.org/taxonomy/10116)]. We filtered peptide annotations to a 1% false positive identification rate. For label-free quantification, we tabulated all unique peptides from matrix and membrane extracts with their chromatographic peak area intensities across all biological replicates (*n* = 8). Data were log_2_ transformed, normalized, and imputed for nonrandom missing values [for more detail, see Supplemental Material, p. 5 (http://dx.doi.org/10.1289/ehp.1104857)].

*Interaction informatics*. Peptide measures found to be statistically responsive to ETS exposure reflected putative modulation of a parent protein or protein family’s abundance, modification, or localization. We performed protein enrichment analysis against Gene Ontology (GO) Annotation terms (biochemical process and cellular component) (GO Consortium, http://www.geneontology.org/) and biochemical pathways using a Fisher’s inverse chi-square method with Bonferroni correction [ToppGene (http://toppgene.cchmc.org/); initial α⊇=⊇0.05] (Chen et al. 2009). Further detail on enriched pathways was assessed through the KEGG Pathway Database (Kanehisa et al. 2010). Proteins associated with the GO term mitochondrion GO:0005739, the most significant enriched cellular component, were analyzed further using protein–protein network analysis [STRING version 8.3 (Szklarczyk et al. 2011)] with the following parameters: a minimum interaction confidence score of 0.5, ≤ 10 interactors, and displayed in evidence view applying a Markov Cluster algorithm.

*Immunoblot analysis*. We resolved protein-balanced samples (10 μg) using the NuPAGE gel system with 4–12% Bis-Tris gels, and MOPS running buffer (Life Technologies, Grand Island, NY). The samples were then transferred to a polyvinylidene fluoride membrane (Millipore, Billerica, MA) via a semi-dry method using NuPAGE transfer buffer (Life Technologies). We then probed the membrane with anti-mouse hexokinase-1 (Hk1) (Sigma-Aldrich, St. Louis, MO), anti-mouse ATP synthase 5A (ATP5A) (Abcam, Cambridge, MA), or anti-rabbit dynamin-1-like (Dnm1l) protein (Origene, Rockville, MD). We used IgG horseradish peroxidase–conjugated secondary antibodies and the SuperSignal West Pico chemiluminescence detection kit (Thermo Scientific) for imaging. Blots were re-probed with anti-mouse β-actin (Abcam) to control for load error. We acquired 16-bit blot images on an Image Station 4000MM Pro imager (Carestream Health, Rochester, NY) and measured net band intensity.

*Immunofluorescence microscopy*. Cerebellar hemisphere tissues were sagitally cryosectioned (10 µm) from 2–2.25 mm lateral of midline and fixed with 3% paraformaldehyde. Sections were probed with anti-mouse mitofilin (MitoSciences, Eugene, OR), anti-rabbit Dnm1l (Origene), and anti-rabbit Calbindin1 (Swant, Marly, Switzerland). We used Alexa Fluor conjugated secondary antibodies (Life Technologies) to acquire at least four images per lamina in lobule VIII from four sections on a Zeiss AxioImager A1 fluorescence microscope (Carl Zeiss, Oberkochen, Germany) using identical parameters. Images were analyzed blind to treatment with relative fluorescent intensity measured for regions of interest using ImageJ software (Abramoff et al. 2004).

*Transmission electron microscopy*. Additional sagittal sections from ETS and control tissues described above were collected at a 50-µm thickness. They were fixed with 2% glutaraldehyde/2% paraformaldehyde (60 min at 4°C), and post-fixed in 1% osmium tetroxide (2 hr). Afterward, sections were dehydrated through a graded ethanol series (50–100%), transitioned through propylene oxide, and then infiltrated overnight in Embed 812 (Electron Microscopy Sciences, Hatfield, PA). We collected thin sections (80 nm) by ultramicrotomy onto copper 300-mesh thin bar grids and contrasted the sections in lead citrate and uranyl acetate. We used a Jeol JEM-1230 transmission electron microscope (Tokyo, Japan) to collect digital micrographs. Morphometric stereological analysis of mitochondria was performed about the medial neurpil of the cerebellar molecular layer (Gosker et al. 2007; Siskova et al. 2010). Measurements included the mitochondrial fractional area (FA), the mean mitochondrial profile area (MA), and the mean number of mitochondria per area (MD). Mitochondrial profiles were counted and circled if they fell within a counting frame grid placed sequential-randomly five times per section as optimized from pilot images in order to measure 100–200 mitochondria per animal (Mouton 2002). We also assessed mitochondrial structure among subpopulations localized within the soman and processes of Purkinje, granular, and glial cells.

*Statistical analysis.* We evaluated locomotor activity using general linear model multivariate testing and least significant difference post hoc comparisons (α = 0.05) in SPSS (version 20; IBM, Armonk, New York). We used one-way ANOVA (analysis of variance) testing on normalized neuroproteomic data using DanteR software (Audinate, Portland, OR) [see Supplemental Material, p. 5 (http://dx.doi.org/10.1289/ehp.1104857)]. We corrected for multiple peptide measures using the Benjamini-Yekutieli false discovery rate method to control type 1 error to 5% (Benjamini and Yekutieli 2001). We loaded normalized immunoblot data in ratio to corresponding β-actin data. Immunoblot and microscopy data were tested using either Student’s *t*-test or a two-way ANOVA with the Holm-Sidak method (α = 0.05).

## Results

**Figure 1:**
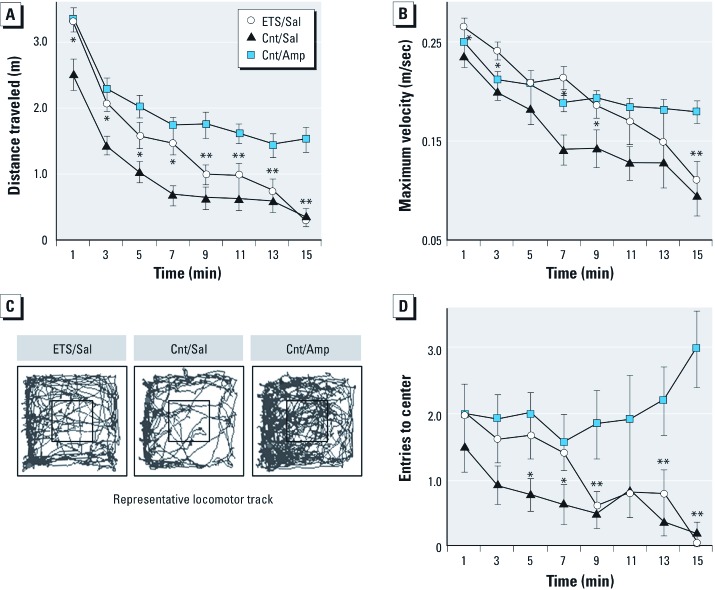
Spontaneous locomotor activity is increased after postnatal ETS exposure. After initial rat cerebellar cortex formation, locomotor activity in a 42 × 42 × 30 cm^3^ open field was recorded at PD25 for ETS_100_-exposed or plain-air control (Cnt) animals. Amphetamine (Amp; 1 mg/kg) as a hyperlocomotion positive control or saline vehicle (Sal) was injected ip 15 min previously. (*A*) Plot of total distance traveled quantified in 1-min bins. (*B*) Plot of maximum velocity quantified in 1-min bins. (*C*) Locomotor track plots for those animals with a median measure of total distance traveled per treatment group; 14 × 14 cm^2^ central zone shown boxed. (*D*) Plot of entries into the central zone quantified in 1-min bins. Data are presented as mean ± SE; *n *= 14/group. *p < 0.05 ETS/Sal compared with Cnt/Sal; **p < 0.05 ETS/Sal compared with Cnt/Amp.

*ETS exposure during postnatal cerebellar development heightens locomotor activity*. Our model of ETS exposure resulted in heightened locomotor activity in a novel environment. We found a significant main effect of ETS exposure for measures of distance traveled (*F*_1,28_ = 26.49, *p* = 1.9E^–5^), maximum velocity (*F*_1,28_ = 8.7, *p* = 0.006) and entries into the center zone of the test arena (*F*_1,28_ = 6.5, *p* = 0.017). Figure 1 displays the time course of habituation to the novel environment and representative locomotor track plots for each group. Post hoc comparisons across time are illustrated as being significantly different between ETS_100_-exposed animals (ETS/Sal) relative to a matched air-exposed group (Cnt/Sal) as well as to an air-exposed group stimulated with amphetamine (1 mg/kg, Cnt/Amp). We used the Cnt/Amp group as a positive control for a heightened locomotor response, which, as expected, maintained a higher asymptotic level of locomotor activity during and after habituation. When placed in a novel environment, ETS/Sal animals initially exhibited a locomotor phenotype resembling that of Cnt/Amp-stimulated animals. While ETS/Sal animals were more reactive (i.e., exhibited more locomotion) to a novel environment than Cnt/Sal animals, they eventually achieved the same baseline as Cnt/Sal animals during the second half of the session.

**Figure 2:**
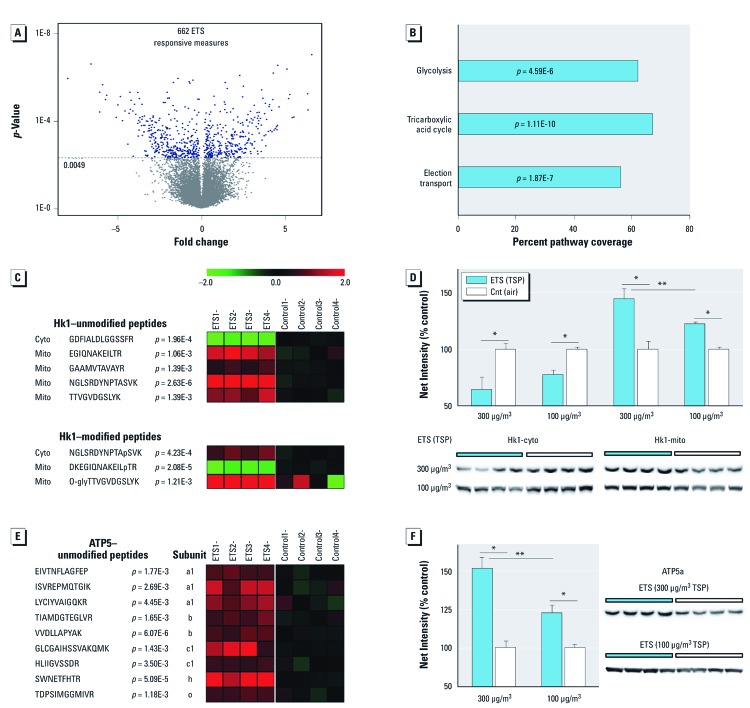
Significant cerebellar proteome perturbation after ETS exposure with dose-dependent up-regulation of aerobic processes. Animals were exposed daily to ETS or plain air (Cnt). (*A*) Quantitative proteomics revealed 662 ETS_300_-exposure responsive peptides in the cerebellar cortex, denoting significant change among 389 proteins; data are presented as fold change (*n *= 4/group; corrected α = 0.0049). (*B*) Aerobic respiration pathways prominently altered in the ETS-responsive neuroproteome; data are presented as percent pathway coverage (corrected α = 0.016). (*C*,*E*) Heatmap plots of ETS_300_-responsive peptide measures for Hk1 (*C*; unmodified and posttranslationally modified) and ATP5 (*E*) (log_2_, ratio to control; *n *= 4/group; *p*-values reported). (*D*,*F*) Dose-dependent immunoblot protein measures of Hk1 (*D*) and ATP5a (*F*); cytosolic (cyto) and mitochondrial (mito) protein levels graphed for Hk1 to assess subcellular translocation; data are presented as mean ± SE; *n *= 4/group. *p < 0.05 compared with controls. **p < 0.01 compared across dose.

*ETS induces underlying cerebellar mitochondrial subproteome perturbation and up-regulates aerobic respiration machinery.* To begin to understand the neurodevelopmental effects of ETS exposure and the underlying mechanisms of action, we employed a systems-based approach beginning with unbiased proteomic analysis. Bioinformatic assessment [see Supplemental Material, Figure S1 (http://dx.doi.org/10.1289/ehp.1104857)] of neuroproteomic change after daily ETS_300_ exposure revealed 662 responsive peptide measures (Figure 2A) that denoted translational and posttranslational dynamics among 389 proteins. This ETS-responsive neuroproteome particularly overrepresented change to the mitochondrial subproteome (103 proteins representing 28% of mitochondrion GO term GO:0005739, *p* = 5.33E–29). All three major aerobic respiration pathways responded to ETS exposure (Figure 2B). This included significant modulation of all glycolytic enzymes, 16 proteins involved in downstream pyruvate processing (e.g., 5 of 8 tricarboxylic acid cycle enzymes), and 10 subunits of four electron transport chain complexes [see Supplemental Material, Figure S2 (http://dx.doi.org/10.1289/ehp.1104857)]. These data revealed that ETS exposure during cerebellar cortex development prominently influenced mitochondria and, in particular, processes involved in aerobic function.

We next assessed the regulational state of aerobic metabolism, which is governed by posttranslational dynamics of the rate-limiting enzyme Hk1. Hk1 peptide measures (Figure 2C) indicated a treatment-induced shift in localization. Mass spectrometry also revealed three previously unknown phosphorylated and glycosylated Hk1 motifs that were responsive to ETS_300_ exposure (Hk1–modified peptides) [Figure 2C; see also Supplemental Material, Figure S3 (http://dx.doi.org/10.1289/ehp.1104857)]. We further affirmed that Hk1 translocation was dose-dependent with 45% and 22% shifts to the mitochondrial membrane relative to controls after ETS_300_ and ETS_100_ exposures, respectively (Figure 2D). We also observed an ETS_300_-induced increase in peptide levels of ATP synthase (ATP5) (Figure 2E), which exhibited a dose-dependent 51% or 23% response to ETS_300_ and ETS_100_ exposures, respectively, over matched controls (Figure 2F).

**Figure 3:**
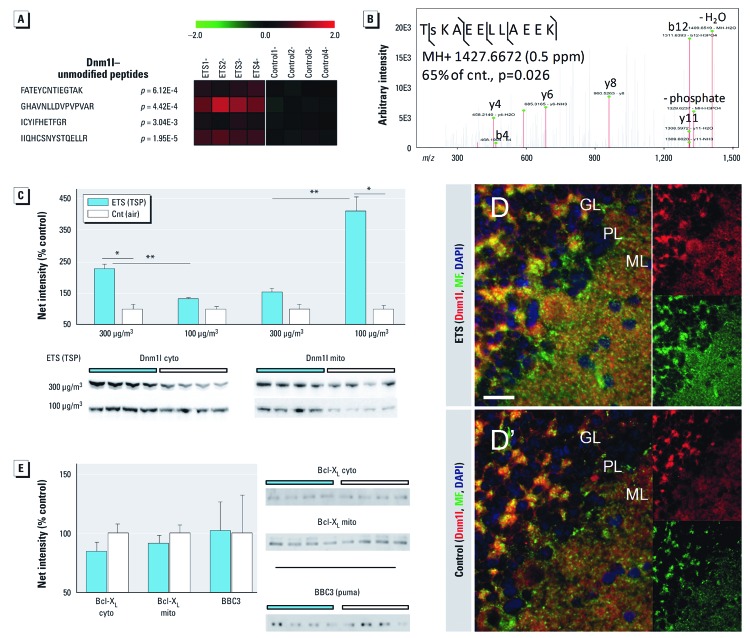
ETS stimulates Dnm1l-mediated mitochondrial fission independent of stress-induced Bcl‑2 family dynamics. Animals were exposed daily to two levels of ETS (300 µg/m^3^ or 100 µg/m^3^ TSP) or plain air (Cnt). (*A*) Heatmap of fission factor Dnm1l unmodified peptide measures within the ETS-responsive neuroproteome. (*B*) Tandem mass spectrum confirming reduced phosphorylation at S_615_ (Log_2_, ratio to control; *n *= 4/group). (*C*) Protein levels of cytosolic (cyto) and mitochondrial (mito) Dnm1l were measured by immunoblot. (*D*,*D´*) Color-coded coimmunofluorescence staining of Dnm1l, the mitochondrial marker mitofilin (MF), and 4’,6‑diamidino-2-phenylindole (DAPI) in cerebellar cortex of ETS_100_-exposed (*D*) and air-control animals (*D´*); granular (GL), Purkinje (PL), and molecular (ML) laminae are demarked, and Dnm1l and MF channels are displayed separately on right; bar = 20 µm. (*E*) Protein levels of Bcl‑2–family members were measured by immunoblot analysis, indicating no significant response to ETS exposure. Data are presented as mean ± SE; *n *= 4/group. *p < 0.001 compared with controls. **p < 0.001 compared across dose.

*ETS stimulates Dnm1l mitochondrial fission independent of stress-induced Bcl2 family dynamics*. We examined whether the significant up-regulation of aerobic respiration machinery co-occurred with altered mitochondrial fission/fusion dynamics. The ETS-responsive neuroproteome revealed significant modulation of the mitochondrial fission factor Dnm1l (or Drp1), whereas the mitochondrial fusion factor mitofusin was unresponsive to ETS exposure. Dnm1l peptide measures reflected a significant increase with ETS_300_ exposure (Figure 3A). We further measured 35% less phosphorylation at S_615_ (S_596_ in human) relative to control (*p* = 0.026) by tandem mass spectrometry (Figure 3B). After ETS_300_ exposure, we measured a robust 125% increase in cytosolic Dnm1l; however, a lower 53% increase in mitochondria-bound Dnm1l did not reach significance at *p* = 0.15 (Figure 3C). In sharp contrast, the milder ETS_100_ exposure induced a 311% increase in active membrane-bound Dnm1l over controls, while the 32% increase in cytosolic Dnm1l was more in line with a dose-dependent effect of exposure. Increased Dnm1l staining was more broadly distributed across molecular (ML) and Purkinje (PL) laminae of ETS_100_-exposed animals relative to controls (Figure 3D and D´, respectively). We further observed a corresponding increase in mitochondrial marker staining (MF), with Dnm1l-stained puncta found adjacent to MF-stained mitochondria. We assayed the Bcl-2 family members Bcl-X_L_ and BBC3, which are known modulators of mitochondrial dynamics and Dnm1l; however, both were found unchanged with ETS exposure (Figure 3E).

**Figure 4:**
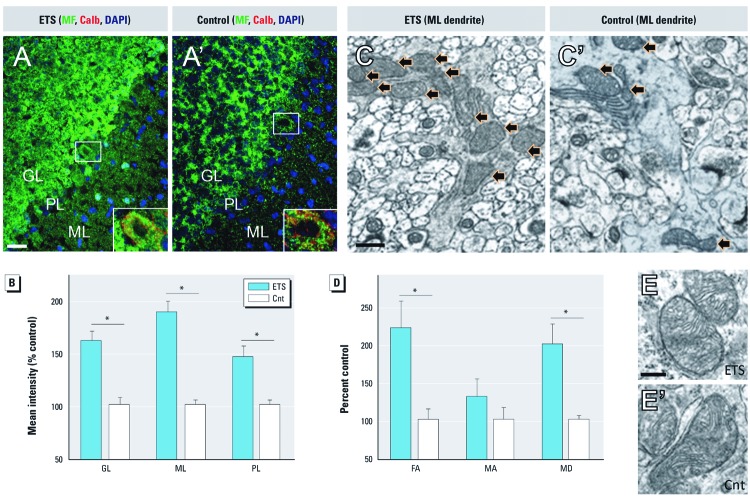
ETS induces Dnm1l-mediated mitochondrial biogenesis, not fragmentation, in cerebellar cortex. (*A*,*A´*) The density of mitochondrial staining (MF) increased in ETS_300_-exposed animals (*A*) relative to air controls (*A´*) throughout cerebellar cortex; color-coded representative images; bar = 20 µm; Purkinje cell staining magnified in inset. (*B*) Mean MF immunofluorescence intensity was significantly greater in granular layer (GL), Purkinje layer (PL), and molecular layer (ML) of cerebellar cortex. (*C*,*C´*) Representative electron micrographs show more mitochondria within ML Purkinje dendrites (black arrows) of ETS-exposed animals (*C*) relative to control (*C´*). (*D*) Stereological measures of mitochondrial profiles within the ML; mean mitochondrial fractional area (FA), mitochondrial profile area (MA), and mitochondrial density (MD). (*E*,*E’*) Mitochondrial ultrastructure appears healthy and consistent between ETS-exposed (*E*) and control (*E’*) animals; representative images from the ML; bar = 200 nm. Data are presented as mean ± SE; *n *= 4/group. *p < 0.01 compared with controls.

*ETS induces mitochondrial biogenesis, not fragmentation, in cerebellar cortex.* We further evaluated the extent and nature of Dnm1l-mediated mitochondrial fission. As previously shown with ETS_100_ exposure, ETS_300_ exposure significantly increased mitofilin-stained mitochondria within the ML and PL (Figure 4A). In contrast, granular layer (GL) mitochondrial staining was also heightened with ETS_300_ exposure. Mean immunofluorescence intensity was significantly greater in all three laminae (Figure 4B), with the greatest increase relative to control in the ML. To explore whether these results were consequent to an increase in mitochondrial fragmentation (degeneration), network size, or biogenesis we examined mitochondrial morphology by electron microscopy. Morphometric stereological analysis affirmed that the fractional area occupied by mitochondria in the ML doubled with ETS_300_ exposure relative to controls (Figures 4D,D´), which closely agreed with our immunofluorescence data. The mean profile area, an indication of mitochondrial size, showed no statistical difference between groups, whereas the count of mitochondrial profiles per field was significantly greater at double that of control. Qualitative assessment of micrographs localized the greater mitochondrial density particularly to Purkinje dendrites in the ML of ETS-exposed animals relative to controls (Figures 4C,C´). Yet, mitochondrial ultrastructure remained consistent and healthy appearing between groups (Figure 4E,E´), with no distinguishable morphological difference from control to suggest stress-induced fragmentation across neuronal and glial subpopulations within the GL, PL, and ML [see Supplemental Material, Figure S4 (http://dx.doi.org/10.1289/ehp.1104857)].

## Discussion

To our knowledge, these are the first findings to demonstrate a significant effect of postnatal ETS exposure on behavior and a potential underlying mechanism involving perturbed mitochondrial energetics critical to the developing brain. This novel insight into the pathobiological impact of ETS during a vulnerable period is the product of a systems-based approach using unbiased proteomic assessment. Prenatal maternal exposure to cigarette smoke has been well documented to induce neurological as well as many other lasting health effects as reviewed elsewhere (Doherty et al. 2009; Pauly and Slotkin 2008). Yet, very few studies have explored neurobiological effects relevant to postnatal ETS exposure, despite mounting evidence for adverse behavioral and cognitive outcomes. Thus, we believe results from this study represent a significant advance in the limited knowledge affirming neurobehavioral and neurobiological effect of ETS exposure during development.

Cerebellar perturbation can broadly impact regulation of behavioral and cognitive domains (Steinlin 2008). Our results show that animals exposed to ETS during postnatal cerebellar development exhibited heightened locomotor activity in a novel environment with a slower rate of habituation relative to controls. The initial heightened locomotor response of the ETS/Sal group was remarkably similar to that of animals injected with a moderate amphetamine dose (Cnt/Amp). These findings were observed across three different dependent measures for the first half of the session. Further, ETS/Sal animals were slower to habituate than either control group, but reached a similar baseline activity to Cnt/Sal animals during the last half of the session. These data suggest an increased response and diminished ability to habituate to an unfamiliar open area rather than a persistent hyperlocomotor response as observed with amphetamine stimulation. Such a behavioral phenotype might result from an ETS-mediated perturbation of inhibitory control loops between cerebellum and neocortex that govern action control (Altman and Bayer 1997). Altman and Bayer (1997) demonstrated that late perturbation during the rat cerebellar vulnerable period, as studied here for ETS, selectively induced (potentially hazardous) heightened activity by impacting late ML synaptic development. These findings stand in contrast to the generalized mobility deficits seen with cell loss after early cerebellar insult.

The functional deficits seen in ETS-exposed children suggest perturbation to circuits involving multiple brain regions, and indeed we previously observed change in the frontal cortex, hippocampus, and cerebellum after modeled adult ETS exposure (Fuller et al. 2010). However, the mechanistic studies here warranted anatomical focus. Cerebellar development was a rational point of investigation given extended postnatal vulnerability and relevance to deficits and disorders impacted by ETS exposure in children. Interestingly, Gospe et al. (1996) in the first neurobiological study to model postnatal ETS exposure showed a greater effect in hindbrain over forebrain, suggesting cerebellar susceptibility. Present findings affirm a significant neurobiological effect of postnatal ETS exposure on the cerebellum, including at household-relevant levels. We further identified perturbed mitochondrial energetics as an important underlying mechanism given the correlation to neuronal activity (Kann and Kovacs 2007).

Aerobic demands increase postnatally with heighted synaptic development, requiring more ATP to maintain membrane polarity. Our results show that developmental ETS exposure perturbed mitochondria and associated aerobic pathways. Hk1 is a key regulator of aerobic ATP production, governed by dynamic recruitment from the cytosol to the mitochondrial membrane (de Cerqueira Cesar and Wilson 2002). Our data reveal a dose-dependent shift in Hk1 to the mitochondrial membrane with ETS exposure. Hk1 translocation involves a positive-feedback mechanism with ATP5 utilizing yet unknown posttranslational signaling to alter Hk1 conformation and binding (Hashimoto and Wilson 2000; Pastorino and Hoek 2008). Our results show a corresponding dose-dependent increase in ATP5 and modification of three previously unreported Hk1 posttranslational motifs in response to ETS exposure. Indeed, these modification sites might be found to govern Hk1 dynamics under aerobic respiration with future research.

Brain energetics is further regulated through mitochondrial fission/fusion dynamics. The ETS-responsive neuroproteome reveals significant up-regulation and modification of fission factor Dnm1l. Predominantly in the cytosol, Dnm1l initiates fission when recruited to the mitochondrial membrane after posttranslational modification (Baloh 2008; Berman et al. 2008; Uo et al. 2009). Dnm1l was significantly dephosphorylated at S_615_, a motif believed to inhibit function, that is, Dnm1l activity is disinhibited after ETS exposure (Corradino and De Palma 2011; De Palma et al. 2010). Greater Dnm1l-stained puncta were found localized with mitochondria, particularly within the ML, with localization to Purkinje dendrites observed by electron microscopy. Heightened ML plasticity critical to cerebellar function remains ongoing through childhood into adolescence (Tiemeier et al. 2010). Interestingly, Li et al. (2008) reported Dnm1l involvement in synaptogenesis as well as mitochondrial biogenesis during development. Our findings may also correlate or perhaps compensate for ETS-altered maladaptive synaptic organization given the close relationship between mitochondrial energetics and synaptogenesis and plasticity in the brain. Thus, the relationship between Dnm1l-mediated mitochondrial biogenesis and aberrant synaptic formation or function after ETS exposure warrants future exploration.

Our results also refute the alternative of oxidative stress–induced mitochondrial fragmentation, which too is mediated by Dnm1l. Cigarette smoke is well known to induce oxidative stress in other organs resulting in mitochondrial dysfunction and a pro-apoptotic environment involving Bcl-2 family signaling (Armani et al. 2009; Westbrook et al. 2010). In particular, Bcl-X_L_ binds and activates Dnm1l fission under oxidative-stress conditions (Wu et al. 2011). Yet, our results demonstrate that Bcl-X_L_ is unresponsive to ETS exposure. Likewise, ETS exposure did not affect levels of pro-apoptotic Bcl-2 binding component 3. Mitochondrial structure appeared undisturbed with ultrastructural analysis. The ETS-responsive neuroproteome lacked a significant association with oxidative stress or apoptotic pathways. Moreover, oxidative stress is known to reduce aerobic respiration, in contrast with our finding of up-regulated aerobic processes. Together, these results support that Dnm1l activation occurs independent of stress-induced Bcl-2 family dynamics and that mitochondrial fragmentation is not occurring in the cerebellum after ETS exposure.

Importantly, we found a dose dependency in the biochemical response to ETS exposure. Most measured changes were halved in response to a 3-fold reduction in ETS levels. These data suggest that a further 3-fold reduction in ETS exposure could still result in a significant effect, assuming a linear relationship, which suggests relevance across a majority of household exposure. Critically, our results show a greater increase in active mitochondrion-tethered Dnm1l after lower ETS_100_ exposure relative to control despite lower overall expression of the protein relative to ETS_300_ exposure. Future studies are needed to demonstrate an effect at lower-level or low-incidence ETS exposure. Also of importance is the interaction between ETS and chronic urban air pollution given prevalent co-exposure. Recently, Calderón-Garcidueñas et al. (2011) showed that severe (heightened TSP) urban air pollution is also a risk factor for attention, language, and learning cognitive deficits, suggesting potential for a synergistic effect.

## Conclusion

In summary, ETS exposure modeled during the postnatal vulnerable period of cerebellar development resulted in a behavioral phenotype and underlying perturbation to mitochondrial energetics in cerebellum that suggest an effect on action control. Our findings further support a biological mechanism involving perturbation to Dnm1l-mediated mitochondrial proliferation during critical postnatal cerebellar development, which presents an opportunity for pharmacological intervention. Ongoing cerebellar development, particularly in the molecular layer, is dependent on tight regulation of mitochondrial dynamics. Our data affirm an association of increased Dnm1l activity with mitochondrial biogenesis rather than fragmentation mediated through Bcl-2 family regulation under oxidative stress. Our findings may also have broader implications for other environmental exposures—given that ETS comprises a wide range of toxic chemicals, heavy metals, and combustion particulate matter—and other neuropsychiatric conditions. More recently, cerebellar dysfunction is also being recognized as involved in schizophrenia and autism (O’Halloran et al. 2012). All together, our findings represent a significant contribution to the limited knowledge on the neurodevelopmental effects of ETS exposure and emphasize a mechanism of action involving cerebellar perturbation. By revealing a plausible biological link with mental health disorders, these findings further encourage efforts to eliminate children’s exposure to ETS. Our results also support the therapeutic potential targeting of mitochondrial dynamics to treat ETS-induced neurobehavioral and cognitive deficits that affect long-term quality of life.

## Supplemental Material

(1.6 MB) PDF
