# Supplementary material for: Exposure of Rats to Environmental Tobacco Smoke during Cerebellar Development Alters Behavior and Perturbs Mitochondrial Energetics
Source: Environ Health Perspect. 2012 Sep 26;120(12):1684–91. doi: 10.1289/ehp.1104857 (PMC3548280; doi:10.1289/ehp.1104857)
Supplement: (1.6 MB) PDF [file ehp.1104857.s001.pdf]

SUPPLEMENTAL MATERIAL

Exposure of Rats to Environmental Tobacco Smoke during Cerebellar Development Alters Behavior and Perturbs Mitochondrial Energetics

Brian F. Fuller[1,2], Diego F. Cortes[1], Miranda K. Landis[1], Hiyab Yohannes[1], Hailey E. Griffin[1], Jillian E. Stafflinger[1], M. Scott Bowers[3], Mark H. Lewis[4], Michael A. Fox[1] and Andrew K. Ottens[1,2]*

[1]Departments of Anatomy & Neurobiology, Virginia Commonwealth University, Richmond, Virginia, USA

[2]Departments of Biochemistry and Molecular Biology, Virginia Commonwealth University, Richmond, Virginia, USA

[3]Department of Psychiatry, Virginia Institute of Psychiatric and Behavioral Genetics, Virginia Commonwealth University, Richmond, Virginia, USA

[4]Department of Psychiatry, McKnight Brain Institute of the University of Florida, Gainesville, Florida, USA

***Corresponding Author:**

Andrew K. Ottens, Ph.D.

Department of Anatomy and Neurobiology

Virginia Commonwealth University, PO Box 980709, Richmond, VA, 23298-0709.

Tel: (804) 628-2972; Fax: (804) 828-9477; Email: akottens@vcu.edu

TABLE OF CONTENTS

I. SUPPLEMENTAL MATERIAL, METHODS

Sample preparation and two-dimensional liquid chromatography-tandem mass spectrometry 3-5

II. SUPPLEMENTAL MATERIAL, REFERENCES........ 5,6

III. SUPPLEMENTAL MATERIAL, FIGURES

A. Figure S1. Neuroproteomics experimental workflow........ 7
B. Figure S2. ETS-responsive neuroproteome........ 8
C. Figure S3. MSMS spectra of post-translation modified Hk1 peptides........ 9
D. Figure S4. Mitochondrial structure following ETS exposure........ 10

I. SUPPLEMENTAL MATERIAL, METHODS

Sample preparation and Two-dimensional liquid chromatography-tandem mass spectrometry.

Tissue was homogenized and incubated (90 min at 4°C) in an aqueous buffer containing Complete Protease Inhibitor Cocktail (Roche Biochemicals) and Phosphatase Inhibitor Cocktails 1 and 2 (Sigma). After centrifuging the supernatant was collected as the Matrix Extract. The pellet was washed and resuspended in more buffer with added triton X-100 (1%), EDTA (5 mM) and DTT (1 mM), incubated as before, and the supernatant collected as Membrane Extract 1. The pellet was washed again and resuspended in 60% of above triton buffer / 40% isopropanol, incubated and the supernatant collected as Membrane Extract 2. Cerebellum lysates (100 µg ea.) were exchanged into 25 mM ammonium bicarbonate, pH 8, with 0.1% Rapigest (Waters) using Millipore Microcon YM-10 filters. 50 µg protein samples were reduced with fresh DTT (5 mM) and alkylated with fresh iodoacetamide (15 mM). Samples were digested overnight with Promega Trypsin Gold (1:100, w/w), then dried and reconstituted in 20 µL of 100 mM ammonium formate (pH 10).

Four fractions (15%, 19%, 28% and 45% ACN: 20 mM ammonium formate, pH 10) were sequentially trapped and gradient eluted: 8% to 30% B, 45 min; to 50% B, 15 min; to 85% B, 1 min; hold 4 min; back to 8% B, 15 min at 300 nL/min in 0.1% formic acid modified water : ACN (B). The MS was operated in V-mode at 10,000 resolving power sampling the [Glu1]-fibrinopeptide B (100 nM) external standard every 30 s for accurate mass correction. Data were acquired via data-independent analysis (Blackburn et al. 2010; Geromanos et al. 2009), m/z 400 to 1,500 precursor ion scan followed by m/z 100 to 1,500 product ion scan at 1 Hz. Daily mass

calibration was followed by analysis of an e-coli tryptic digest standard for quality control assessment of system performance.

Data were processed and searched using Waters PLGS software (v.2.4) as detailed elsewhere (Li et al. 2009). Ion data were first tabulated by monoisotopic reduced mass (MH+), peak apex retention time and the combined chromatographic peak area for all isotopes and charge states, producing one ion table per protein extract, per biological sample. Each ion table was parsed against a UniprotKB Rattus Norvegicus specific fasta database (2010_06 release with 15,083 entries) combined with its reversed-sequence decoy entries. Search engine parameters selected for trypsin specificity, one missed cleavage, fixed carbamidomethyl cysteine modification, variable methionine oxidation, neutral loss of ammonia or water, MS tolerance of 10 ppm and MSMS tolerance of 25 ppm. Serial searches were performed considering: without additional post-translational modifications, with variable phosphorylation and then variable glycosylation.

Ion tables for each sample (n=8) were aligned for the Matrix Extract data and Membrane Extract data by precursor ion MH+ and retention time utilizing the PLGS Expressions software described elsewhere (Silva et al. 2005). Note ion data from Membrane Extracts 1 and 2 were combined first given a common sub-cellular origin. Unique peptide measures were sorted by peptide score and filtered to an estimated 1% false sequence identification rate using a decoy database method. Aligned ion tables were filtered to select peptide chromatographic peak area intensity measures quantified in ≥ 75% of biological replicates. Peak area intensity values were collapsed when split by the experimental events missed-cleavage, methionine oxidation and neutral loss, leaving a single, biologically-relevant measure under the MH+ and sequence of the most massive entry (phosphorylated or glycosylated sequences remained separate). All entries

were defined by a unique identifier comprised of the peptide sequence, the integer MH+ value and the protein extract of origin. Data were log2 transformed and normalized (linear model) using DanteR software (v.1.0.1) (Polpitiya et al. 2008). Non-random missing values symptomatic of a group-specific divide about the analytical detection limit were identified, and values imputed per a simulated Gaussian distribution about the detection limit as described previously (Chich et al. 2007).

II. SUPPLEMENTAL MATERIAL, REFERENCES

Blackburn K, Mbeunkui F, Mitra SK, Mentzel T, Goshe MB. 2010. Improving protein and proteome coverage through data-independent multiplexed peptide fragmentation. J Proteome Res 9:3621-3637.

Chich JF, David O, Villers F, Schaeffer B, Lutomski D, Huet S. 2007. Statistics for proteomics: Experimental design and 2-DE differential analysis. J Chromatogr B Analyt Technol Biomed Life Sci 849:261-272.

Geromanos SJ, Vissers JP, Silva JC, Dorschel CA, Li GZ, Gorenstein MV et al. 2009. The detection, correlation, and comparison of peptide precursor and product ions from data independent LC-MS with data dependant LC-MS/MS. Proteomics 9:1683-1695.

Li GZ, Vissers JP, Silva JC, Golick D, Gorenstein MV, Geromanos SJ. 2009. Database searching and accounting of multiplexed precursor and product ion spectra from the data independent analysis of simple and complex peptide mixtures. Proteomics 9:1696-1719.

Polpitiya AD, Qian WJ, Jaitly N, Petyuk VA, Adkins JN, Camp DG,2nd et al. 2008. DAnTE: A statistical tool for quantitative analysis of -omics data. Bioinformatics 24:1556-1558.

Silva JC, Denny R, Dorschel CA, Gorenstein M, Kass IJ, Li GZ et al. 2005. Quantitative proteomic analysis by accurate mass retention time pairs. Anal Chem 77:2187-2200.

III. SUPPLEMENTAL MATERIAL, FIGURES

Figure S1. Neuroproteomics experimental workflow — Page 7

Figure S2. ETS-responsive neuroproteome — Page 8

Figure S3. MSMS spectra of post-translation modified Hk1 peptides — Page 9

Figure S4. Mitochondrial structure following ETS exposure — Page 10

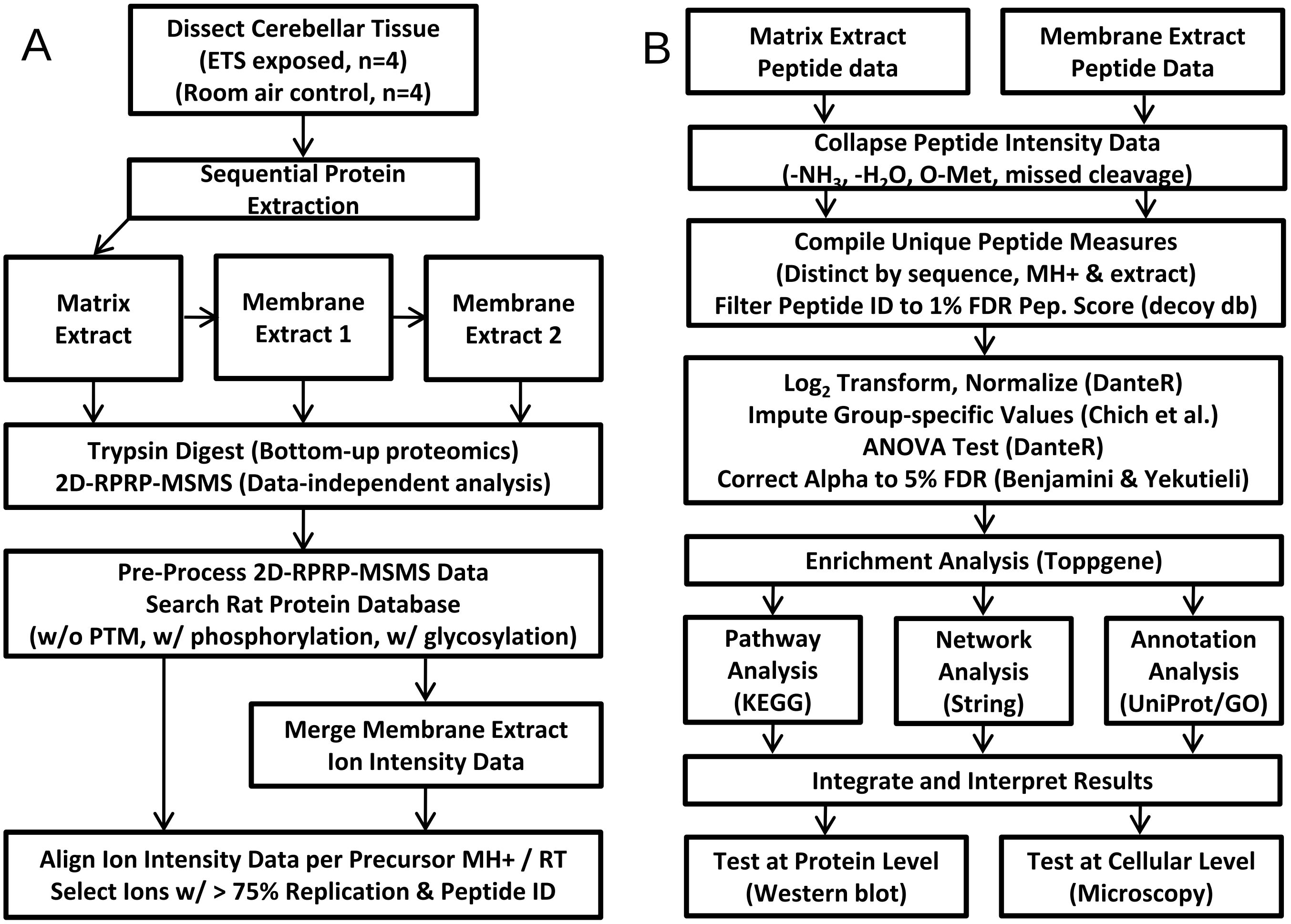

Supplemental Material, Figure S1. Neuroproteomics experimental Workflow. (*A*) Schematic of sample processing and ion data generation steps. Replicate samples were processed using sequential protein extractions followed by two-dimensional reversed-phase tandem mass spectrometry (2D-RPRP-MSMS) of tryptic digests. Ion data were searched against a rat protein database, aligned across all biological replicates and filtered for within-group reproducible measures. (*B*) Schematic of bioinformatics performed. Ion data were post-processed to collapse biologically redundant ion measures, normalize ion area measures, impute for non-random missing values and perform statistical testing. Results were assessed by enrichment, pathway and network analyses, with biological interpretation substantiated by orthogonal molecular and cellular measures.

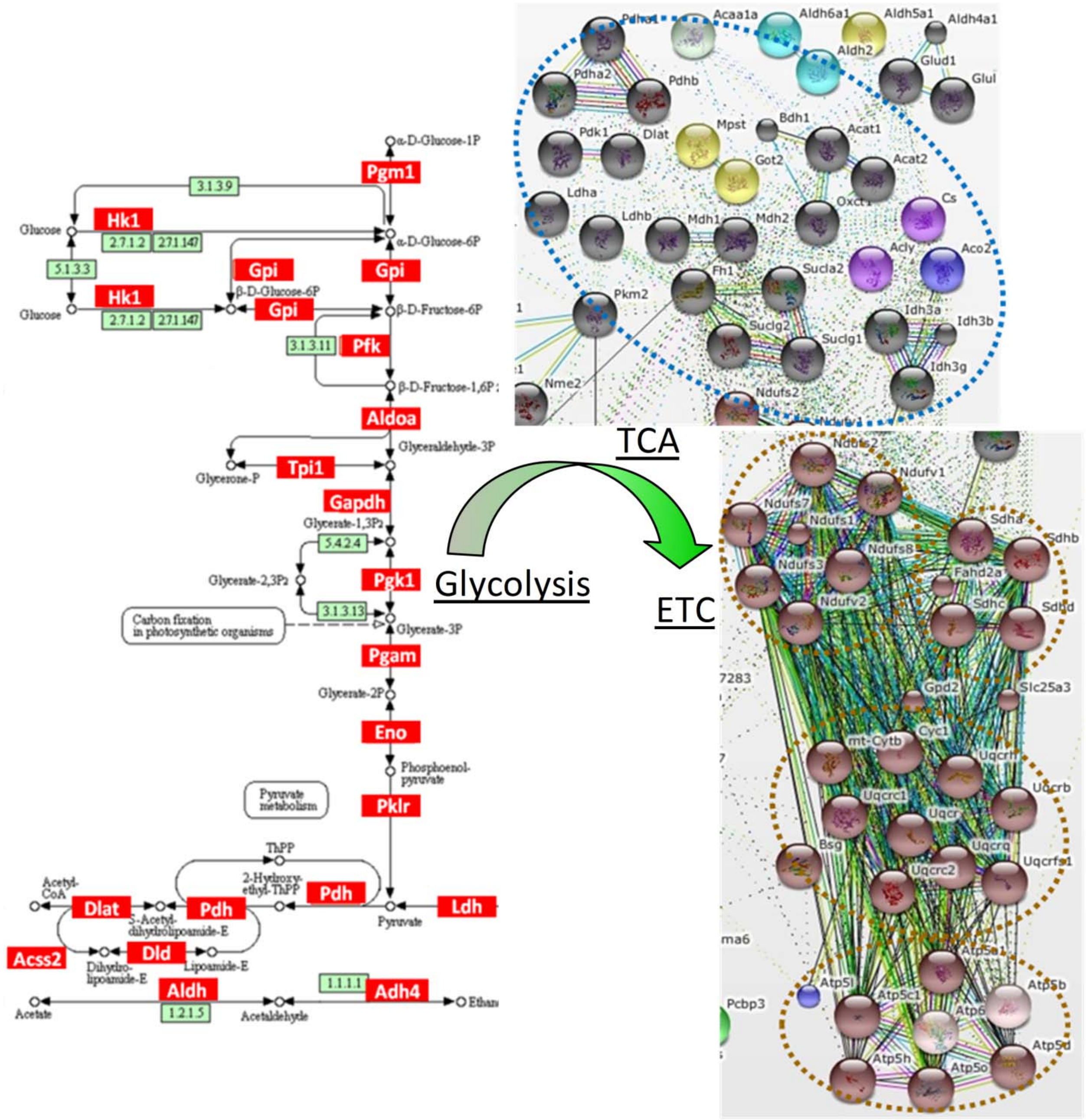

Supplemental Material, Figure S2. ETS-responsive neuroproteome significantly associated with pathways of aerobic respiration. Bioinformatic assessment of the ETS-responsive neuroproteome revealed an overrepresentation of mitochondrial proteins and enrichment for glycolysis, tricarboxylic acid cycle (TCA) and electron-transport chain (ETC) pathways involved in aerobic respiration. At left is an illustration of the overlap with glycolysis, with responsive proteins boxed in red. In the center is a large cluster of proteins associated with pyruvate processing and the TCA cycle. At right are 4 protein clusters representing the ETS-responsive subunits associated with ETC complexes 1, 2, 3, and 5.

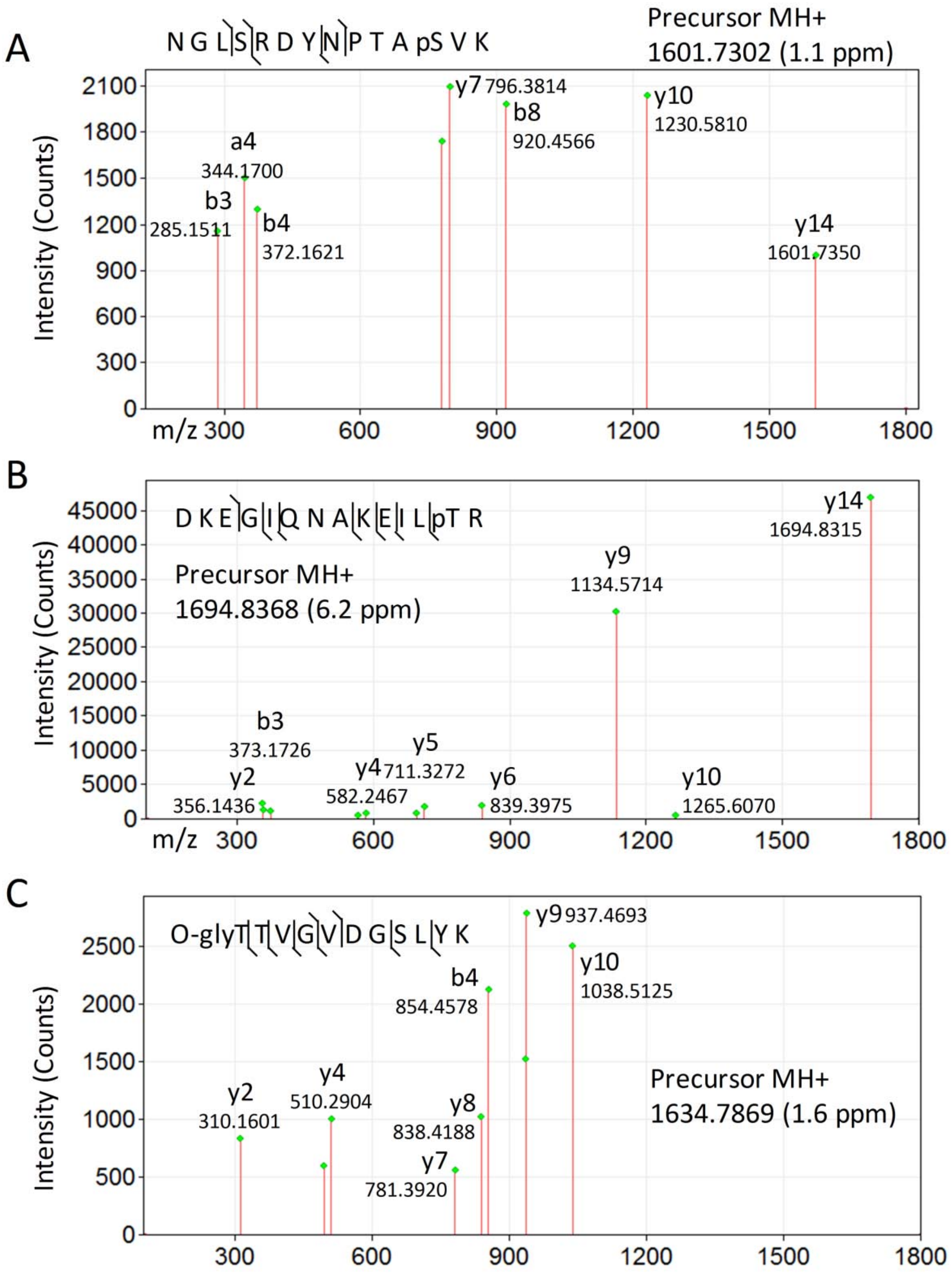

Supplemental Material, Figure S3. MSMS spectra of post-translation modified Hk1 peptides. Data-independent MSMS spectra for two phosphorylated (*A & B*) and one O-linked glycosylated (*C*) peptides of Hk1. Data were deisotoped and mass reduced to a single charge state per normal PLGS processing. Data-independent MSMS spectra included product ions from co-eluting parent ions, which are resolved by accurate mass measures and time alignment. Measured precursor MH+ (ppm error) values are indicated. Product ions matched to the sequence within a 100 ppm tolerance are highlighted in red and annotated by their fragmentation series position. Product ions in (*A*) indicated phosphorylation at either position 10 or 12 and in (*B*) at residue 13. The N-terminal glycan in (*C)* was matched to $Deoxyhex_2HexNAc_1$ (495.195 amu modification).

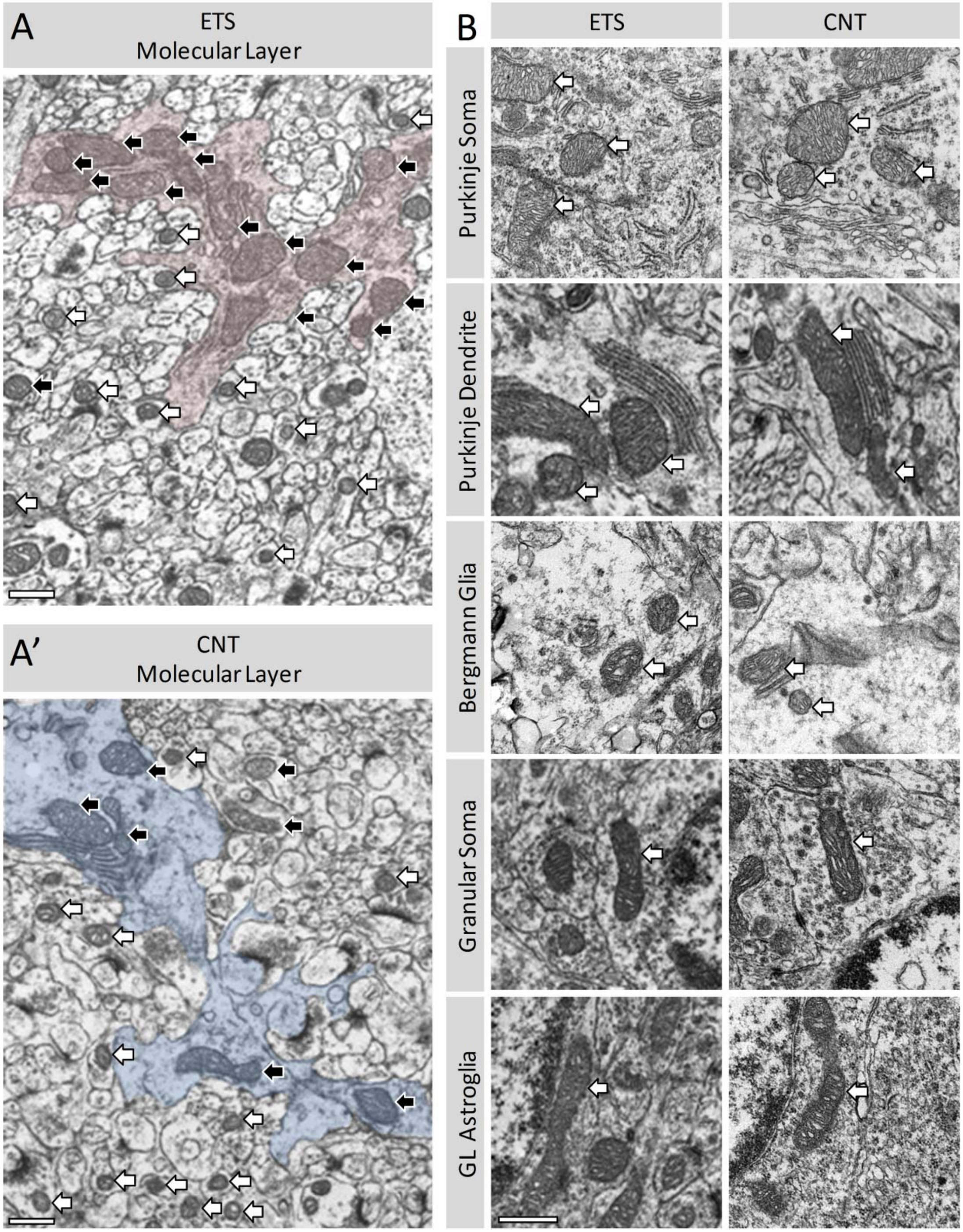

Supplemental Material, Figure S4. Mitochondrial structure following ETS exposure. Mitochondrial profiles exhibited similar ultrastructure between ETS (A) and control (A') groups across dendritic (in blue) and axonal elements in the ML. An ETS-induced increase in the mitochondrial area fraction quantified in ML (see Fig. 4) appears to correspond with a greater density observed within dendritic (black arrows) rather than parallel fiber (white arrows) projections, though the later are difficult to distinguish from dendritic spines. Mitochondria appeared structurally similar between groups among sub-populations localized to specific cellular compartments (B). Representative images; bars = 500 nm (bar in B representative for entire panel).
